# Generating synthetic genotypes using diffusion models

**DOI:** 10.1093/bioinformatics/btaf209

**Published:** 2025-07-15

**Authors:** Philip Kenneweg, Raghuram Dandinasivara, Xiao Luo, Barbara Hammer, Alexander Schönhuth

**Affiliations:** AG Machine Learning, Bielefeld University, Bielefeld, NRW 33615, Germany; AG Genome Data Science, Bielefeld University, Bielefeld, NRW 33615, Germany; College of Biology, Hunan University, Hunan, Hunan Province 410082, China; AG Machine Learning, Bielefeld University, Bielefeld, NRW 33615, Germany; AG Genome Data Science, Bielefeld University, Bielefeld, NRW 33615, Germany

## Abstract

**Summary:**

In this paper, we introduce the first diffusion model designed to generate *complete* synthetic human genotypes, which, by standard protocols, one can straightforwardly expand into full-length, DNA-level genomes. The synthetic genotypes mimic real human genotypes without just reproducing known genotypes, in terms of approved metrics. When training biomedically relevant classifiers with synthetic genotypes, accuracy is near-identical to the accuracy achieved when training classifiers with real data. We further demonstrate that augmenting small amounts of real with synthetically generated genotypes drastically improves performance rates. This addresses a significant challenge in translational human genetics: real human genotypes, although emerging in large volumes from genome wide association studies, are sensitive private data, which limits their public availability. Therefore, the integration of additional, insensitive data when striving for rapid sharing of biomedical knowledge of public interest appears imperative.

**Availability and implementation:**

All non proprietary data and the code to replicate the experiments is available on Github.

## 1 Introduction

Deep learning has enabled significant advancements in the field of computational biology (Jumper *et al.* 2021, Cheng *et al.* 2023, Wong *et al.* 2024). However, the vast majority of such approaches resort to processing smaller portions of human genomes, such as coding regions and their products (proteins), or small local segments of human genomes (Shen *et al.* 2022, Guo *et al.* 2024).

The reasons for this are 3-fold. First, the enormous length of human genomes prevents their straightforward usage in neural network architectures (Nguyen *et al.* 2023). Second, whole human genome data are expensive, because of the massive clinical and experimental efforts required in order to obtain them (Schwarze *et al.* 2018). Third, whole human genome data are generally subject to strict access regulations due to privacy concerns (where Project MinE (pro, Project MinE ALS Sequencing Consortium 2018) is one example). The difficulty to process, gather and share sufficient (training) data impedes scientific progress through reliable extraction of knowledge, rapid dissemination of relevant data, and render easy and full reproducibility of already obtained results impossible.

While the first reason is a (challenging) technical concern, the second and the third reason establish our key motivation from the point of view of applications in biomedicine. The solution that we suggest here is the generation of synthetically generated whole genome data that is cheap, easy to gather, and privacy-enhancing. We present a diffusion model based framework that can generate synthetic whole-genome human genotype data.

Despite the relatively small amounts of data used during training, we ensure that our diffusion models do, in fact, *generate novel whole genome human genotypes.*

By means of approved reliable metrics, we ensure that the synthetically generated genotypes are of high quality, that is realistic in terms of stemming from the distribution governing real human genotypes, and also diverse, that is they do not reproduce the individual genomes used for training the diffusion models, which translates into preservation of privacy in the setting at hand.

Note that, unlike previous work (Guo *et al.* 2024), whole-genome genotypes can be straightforwardly expanded into whole DNA-level genomes, by means of applicable genome reference systems. Furthermore, diffusion models enable us to generate disease-affected and non-disease-affected genotypes in a targeted manner. Beyond demonstrating that the synthetically generated genomes are realistic by the measures that ensure that the diffusion model approximately captures the distribution of human genotypes, we further demonstrate that training classifiers (Luo *et al.* 2023) using synthetically generated data, by either integration or replacement, and evaluating them on the original data achieves performance rates that rival those of the original classifiers. has not only captured the general structure of human genomes, but also has picked up the mechanisms that distinguish diseased from non-diseased genotypes.

## 2 Related work

### 2.1 Previous models

In Table 1, we systematically compare most relevant prior work that has tried to produce genomes. To the very best of our knowledge, we are the first approach that succeeds in generating full-length human genotypes. After thoroughly and carefully revisiting the landscape of existing tools and approaches (see Table 1), we conclude that there are indeed no approaches that can generate synthetic full-length human genotypes (or even human genomes directly at the level of DNA). All approaches presented so far address generating smaller segments of human genomes, the majority of which does not even span the length of one chromosome. However, genome-wide association studies or classifiers as the one presented in Luo *et al.* (2023) require full-length human genome data to work well.

**Table 1.** An overview of related work on generating synthetic genomes and its differences/similarities in comparison with our work.^a^

Reference	Model	Data type	Genome length	Cond.
DNAGPT (Zhang *et al.* 2023)	Autoregressive	Base-Pairs	24k BPS	x
HyenaDNA (Nguyen *et al.* 2023)	Autoregressive	Base-Pairs	$10^{6}$ BPS	x
HAPNEST (Wharrie *et al.* 2023)	LD & Markov	SNPs	1 Chromosome	x
Perera *et al.* (2022)	GMMNs	SNPs	1 Chromosome	✓
Yelmen *et al.* (2021)	GAN, RBM	SNPs	10k SNPs	x
Yelmen *et al.* (2023)	WGAN	SNPs	10k SNPs	x
Szatkownik *et al.* (2024)	WGAN	PCA+SNPs	65k SNPs	x
Ahronoviz and Gronau (2024)	GAN	SNPs	10k SNPs	✓
Burnard *et al.* (2023)	VAE	SNPs	1 Chromosome	x
Dang *et al.* (2023)	HCLTs	SNPs	10k SNPs	x
GeneticDiffusion (Ours)	**Diffusion**	PCA+SNPs	**Full Genome**	✓

a Row headers are: Reference, the modeling approach used, the data type the model works on, length of generated genomes presented, whether or not the model can be conditioned to produce specific types of data. Our novelties are **highlighted**.

### 2.2 Generative models

We prefer diffusion models over alternative generative deep learning models for the following reasons: (i) Diffusion models have become the tool of choice in generative modeling (Dhariwal and Nichol 2021), and (ii) previous successes in the use of diffusion models in regulatory human genomics (Avdeyev *et al.* 2023, Li *et al.* 2024, Sarkar *et al.* 2024, Senan *et al.* 2024) further support their usage. Unlike these works, we emphasize that in our work we make use of full-length genotypes, and do not have to restrict ourselves to smaller portions of the human genome. We considered skipping steps for generation as done in DDIM (Song *et al.* 2021) as a way to speed up generation, but realized that the high quality generated by using the full step length for our generation was important for our use case. Furthermore, we considered classifier free guidance (Ho and Salimans 2022) to increase the quality of conditioning during generation, as demonstrated in Azizi *et al.* (2023), but observed no positive effects for classification accuracy on the test data.

### 2.3 Working with long sequences

One of the driving problems when working with genetic data is the enormous length of the genomes. This is exacerbated by the long range interactions that affect parts of the genomes that are far apart in terms of the sequential order in which they appear.

It is therefore no surprise that recent genomics research is using techniques that can accommodate the large length of human genomes (e.g. Nguyen *et al.* 2023, HyenaDNA), which are powerful architectures that are able to process up to a million tokens simultaneously via an adapted form of attention, and also, as of most recently, Caduceus (Schiff *et al.* 2024) based on state space models, for which similar principles apply. However, even with those recent methodological advances, sequences of billions, and not just millions in length, are difficult to process. In the domain of diffusion models, works have been presented that analogously adopt the paradigm of no longer working directly on the raw data, but rather on appropriately embedded versions of it (Rombach *et al.* 2022, “Stable Diffusion”). We adopt this paradigm.

### 2.4 Privacy of synthetic data

In this section, we review common strategies and to ensure privacy when generating synthetic data and contextualize our work from this point of view.

*Definition of privacy:* In the context of genetic data, we consider a synthetic dataset private if it prevents the reconstruction and identification of any individual samples from the original, privacy-protected dataset that serves as the foundation for its generation.

*Threat model:* We propose that synthetic data could be safely published if the generative model itself is withheld. The scenario reflects the existence of large databases that are subject to strict access control. Administrators of such databases train the diffusion model, and provide samples on demand. Note that this does neither mean that parameters of the generative model are to be shared, nor to query the underlying database in alternative ways. By withholding the generative diffusion model and the data that supported its construction, common attacks such as Membership Inference Attacks (MIA) aimed at identifying specific samples, become significantly less feasible (Duan *et al.* 2023). In particular, e.g. differential privacy-type queries are impossible.

*Limitations of privacy protections:* It is important to emphasize that our approach does not provide statistically quantified protection mechanisms in the style of the theory of differential privacy—note however that, in our case, differential queries are impossible anyway, because the database holding the true data remains entirely inaccessible (see above). This means that the attempt to infer individual data points reduces to guessing the empirical data by generating samples from the distribution that governs the empirical data. In our case, this means that one has to overcome two layers of challenges: first, the reconstruction of the diffusion model from samples generated by it (for which, as of today, there is no known procedure), and second, to infer the training data from which the diffusion model was established. The latter amounts to a classic membership inference attack. This, although theoretically not impossible, is a tough challenge in the context of diffusion models.

*Differential privacy for diffusion models (Dockhorn et al. 2023):* Differential privacy can be implemented by introducing calibrated noise throughout the diffusion process, ensuring that synthetic data cannot be traced back to individual real data points. However, adhering to this noise injection approach is known to substantially reduce the utility of generated samples in the bioinformatics context see, Fredrikson *et al.* (2014). In particular, ensuring differential privacy is near-pointless according to our threat model, because database queries are impossible.

*Nearest neighbor adversarial accuracy (NNAA) (Yale et al. 2020):* In this work, we use NNAA, as an established metric to measure the effectiveness of an adversary in distinguishing between in-distribution and out-of-distribution data points based on their nearest neighbors. It allows for the detection of potential replicas of original data within synthetic datasets produced by diffusion models. Note in particular that our threat model allows to prevent the accidental release of individual data records: we can evaluate any sample generated by the model by the metric, and suppress its release if judged to be too close to any individual record by the metric.

## 3 Methods

In the following, we describe the data representations that reflect human genomes, i.e. the genotype profiles that correspond to them, and the computation of embeddings for these genotype profiles, as well as the architectural choices for the diffusion model. For more details on diffusion models and the human Genotype, see the Appendix in the Supplementary Material.

### 3.1 Data

We deal with two datasets of human genotypes:

#### 3.1.1 ALS data

The individual genotype profiles that we use for training (and testing) in the following, were raised in the frame of Project MinE pro (Project MinE ALS Sequencing Consortium 2018), which is concerned with the study of amyotrophic lateral sclerosis (ALS). As a disease, ALS is of particular interest to AI based applications, because ALS is driven by complex, still insufficiently understood mutation patterns that escape the grasp of human-understandable approaches. For exactly these reasons, also earlier studies (Auer *et al.* 2012, Dolzhenko *et al.* 2017) focus on ALS, using data gathered through Project MinE.

While Project MinE establishes a data resource that is exemplary in terms of size and comprehensiveness, access to its data is subject to strict safety regulations. This is the reason why we exclusively deal with a Dutch cohort of people, for which we were provided access, while not with cohorts of genotypes referring to other countries. The Dutch cohort we worked with consisted of 3292 individuals affected by ALS and 7213 individuals known not to be affected by ALS, by ancestral relationships.

Accordingly, for this data, labels *y* used to steer the generation of new samples, refer to individuals affected with ALS and without ALS.

#### 3.1.2 1000 genomes (1KG)

We also consider the 2504 individuals sequenced in Stage 3 of the Auton *et al.* (2015). For these 2504 individuals, alleles were assigned to ancestors (referred to as “phased” in genetics), such that one obtains two *haplotypes* instead of one genotype for each individual, where a haplotype is a binary-valued vector of length *N* where 0 reflects that the reference allele applied at the particular position whereas 1 reflects to observe the alternative allele at the respective SNP site. Adding up the two haplotypes, entry by entry, results in the genotype of the individual. Because, unlike genotypes, the haplotypes assign alleles to ancestors, they carry more information. This is considerably more valuable for genetics, because they provide immediate insight into the ancestral relationships affecting genomes.

When dealing with 1KG data, we seek to generate haplotype profiles instead of merely genotype profiles. Unlike with Project MinE, no particulars about the corresponding phenotypes are known in the frame of the 1KG project. The only known phenotype is the population from which they stem. So, the additional conditioning input *y* refers to these 26 population labels when working with 1KG data.

### 3.2 Embedding genotypes/haplotypes

**Figure 1.**
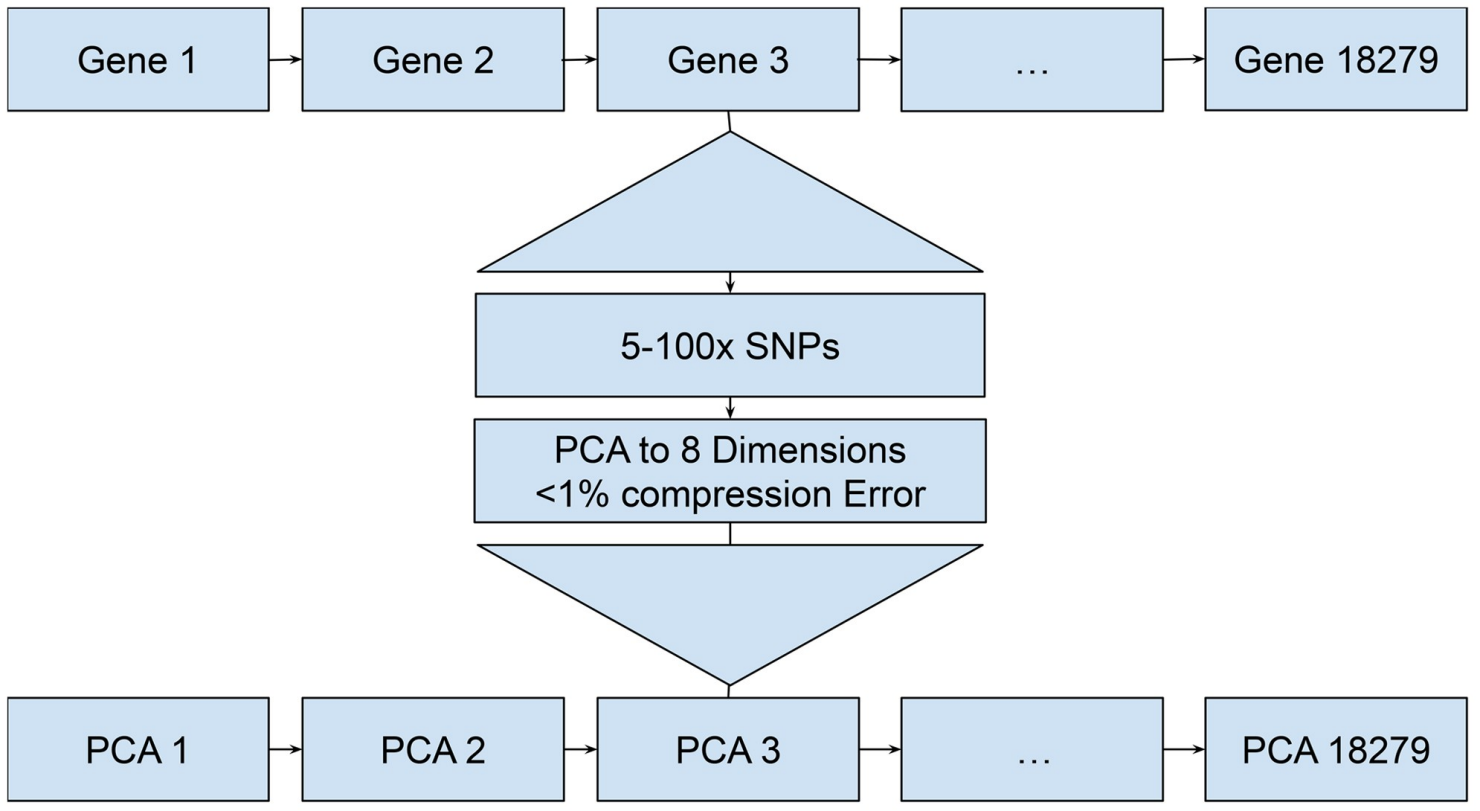
Overview of the pre-processing pipeline. Genes, which consist of between 5 and 100 SNPs are each processed by a custom PCA. This is done independently for each Gene.

In the following, refer to Fig. 1 for an illustration the embedding procedure. See Luo *et al.* (2023) as well as the Appendix, for more details on the following.

Embedding refers to turning ternary-valued (genotypes, ALS) or binary-valued (haplotypes, 1KG) vectors of length approximately 3–5 millions into real-valued vectors, whose dimension is in the tens of thousands. This does not only reduce the dimensionality of the data, but also ensures consistency in terms of arranging the data for appropriate processing by the diffusion model.

For that transformation, we consider the genes recorded for the ALS and 1KG datasets, amounting to 18 279 and 26 624 genes, respectively. Based on approved principles, we assign each SNP site to one of the genes. The number of SNP sites per gene can vary quite substantially. Depending on length and location in the genome, a gene can collect roughly between 5 and 100 SNP sites. In other words, each gene reflects a ternary- (ALS) or binary-valued (1KG) vector of length between 5 and 100. Following the approach described in Luo *et al.* (2023), we apply principal component analysis (PCA) to each of the 18 279 (ALS) or 26 624 binary-valued (1KG) vectors of length between 5 and 100, for each gene separately, see Fig. 1. This amounts to 18 279 (ALS) resp. 26 624 (1KG) PCAs, each one applied to the vector segments referring to one of the genes. The result is a collection of principal components for each of the genes, both in the case of ALS and the case of 1KG, see the bottom line of PCAs in Fig. 1. Depending on the number of SNP sites per gene, we pick between 1 and 8 principal components (PCs) for each of the 18 279 (ALS) or 26 624 (1KG) genes. For consistency and due to architectural reasons, we pad any such vector of length <8 (corresponding to <8 PCs for the particular gene) with zeros to extend it to length 8.

For both ALS and 1KG, the reduction of dimension comes at minimal compression loss (<1%) as shown in Luo *et al.* (2023). This implies that one can decompress the PC embedded data into the original genotypes or haplotypes at negligible losses of information. Observing that 18 432=2^11^ × 9, we further pad embeddings $x\in R^{18279\times 8}$ with further zeros, extending individual genotype embeddings into elements of $R^{18432\times 8}$, which is required for the U-Net architecture. To remedy the issue that zero padded position add additional failure points all values at padded positions are clamped to zero during training and the generation process.

We apply the same procedure for the 26624×8D vectors referring to the 1KG data.

### 3.3 Model architecture

We base our Diffusion Model on the popular U-Net Architecture suggested by Ronneberger *et al.* (2015), while applying modifications that account for the sequential nature of the genetic data. We note that the order of the genes along the genome implies a natural order of the different 8D gene vectors.

We explore different variants of the basic U-Net architecture by replacing the 2D convolutional layers with their 1D counterparts, or with multi-layer perceptrons (MLPs).

**Figure 2.**
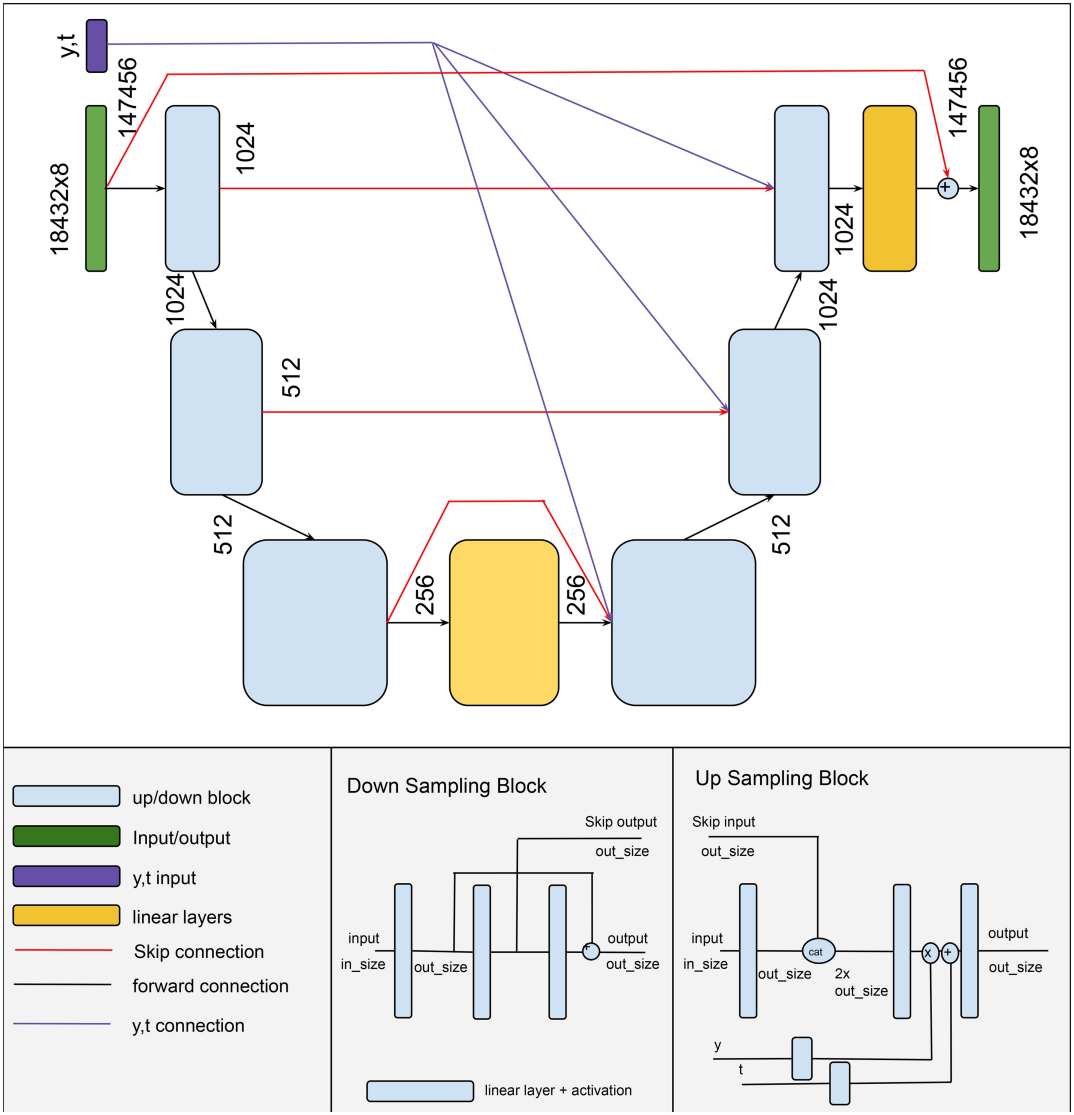
A structural overview of the architecture of the MLP diffusion model. The input is passed through several down sampling and up sampling blocks. Each down sampling block has a skip connection to its respective up sampling counterpart. Conditioning *y*, *t* is injected in the up projection blocks.

We also explore a transformer encoder structure similar to the one presented in Devlin *et al.* (2019), but with learnable positional embeddings, and two additional tokens accounting for time steps *t* and class labels *y*, further equipped with an embedding layer similar to the one used for images in Dosovitskiy *et al.* (2021). This serves the purpose of reducing the number of input tokens which is essential for reducing the computational cost. In Fig. 2, we visualize the UnetMLP architecture that we propose: it is similar to the convolutional Unet, but does not include multi-headed attention in the intermediate layers. For visualizations of the Transformer and UnetCNN architectures see the Appendix in the Supplementary Material.

In general, we want to point out that the approach using 1D convolutions prioritizes short- to medium-length interactions within the genome, while capturing only limited amounts of long-range interactions. On the other hand, this greatly reduces the amount of parameters to be learned, which offsets the disadvantages from a practical point of view. However not including any kind of spatial information does lead to problems on the presented task, due to the high sensitivity of information with regards toward the position. That is, it is highly important that the model has positional information about the PCA it processes. This is not the case for the CNN architecture.

In contrast, models solely incorporating dense layers are not subject to sequential biases. However, as in other domains of applications of neural networks, fully connected layers tend to fail to find suitable solutions due to the over-parameterization (Zhang *et al.* 2019).

Fully attention based models, e.g. models based on transformer encoder architectures do not have any inherent spatial bias, the positional encoding used induces the kind of bias (Vaswani *et al.* 2017). Following, they should be in theory applicable for this kind of data. Therefore, we also explore such architectures here.

#### 3.3.1 Combining models

Since CNN and MLP based models focus on different aspects of the structure of the genome, we suggest combining them into a single network that benefits from the strengths of the two architectural choices, and synthesizes their advantages. We refer to this combination as CNN + MLP. We combine the two separate models MLP(x,t,y) and CNN(x,t,y) during training, and predict the noise (that the diffusion model has added to the input) accordingly:


(1) MC(x,t,y)=(1−λ(t))·MLP(x,t,y)+λ(t)·CNN(x,t,y)

where λ(t),t∈[0,1] reflects a learnable function, realized by a straightforward 2-layer-perceptron receiving the noise schedule *t* as input. And MC refers to the MLP + CNN model.

Overall, we explore 4 different diffusion model architectures: “Unet MLP,” “Unet CNN,” “Unet MLP + CNN,” and “Transformer.” We perform extensive hyper-parameter tuning on all of these model types and present the best results in the evaluation.

### 3.4 Evaluation

Evaluation of synthetically generated genomes requires careful consideration. The driving underlying principles are realism, on the one hand, and diversity on the other hand. While realism refers to synthetic genomes being likely to stem from the true distribution of genomes, diversity is concerned with sampled synthetic genomes being sufficiently far away from the true training data points. In image generation common scores to measure these metrics are the Fréchet inception distance (FID) (Heusel *et al.* 2017) or, the Inception score (Salimans *et al.* 2016), e.g. While human eyesight does not apply in genomics for obvious reasons, the FID and IS cannot be computed either because of the integration of pre-trained large scale (here in particular: inception) networks. In fact, this scenario does not apply in genomics/genetics for exactly the reasons that motivate our work: the lack of available (accessible) large-scale data hampers conventional ML practice. Due to these reasons we are forced to rely on metrics which while proven are a bit more unconventional for the image generation domain namely: Adversarial Accuracy and Classifier Performance.

We note that all datasets we have access to are, although fairly large from the point of view of biomedicine, considerably limited in terms of ML concerns (number of samples at most 10 405). So, one cannot expect the diffusion model to perform at the level of realism observed in other complex domains, such as images and text. This explains why the evaluation relates to exploring the upper limits of possibilities in our context. Note however that our approach virtually serves the purpose of training diffusion models on large-scale data, as hosted by large, access restricted databases, in a safe, access-restricted environment. These databases could then provide safe, privacy-preserving large-scale synthetic data on demand, by drawing samples from the diffusion model, without having to publish neither real data nor the diffusion model trained on real data.

#### 3.4.1 Recovery rate

Using synthetic data for training reflects using samples from a distribution that was estimated using empirical data. Since all knowledge captured by that distribution stems from the real, empirical data from which it was estimated, any samples drawn from that distribution can, at most, convey the knowledge that contributed to its estimation. In terms of classifier performance, this means that performance rates achieved when using real data for training establish an upper bound for the performance rates that one can achieve when using synthetic data which was generated by observing the same real data for training.

More formally, consider a classifier *C* and a generator *G*, both of which are trained on the same real data $D_{r}$. While *G* explicitly tries to approximate the distribution that governs $D_{r}$, *C* implicitly approximates it in order to establish sufficiently accurate classification boundaries. Sampling synthetic data corresponds to drawing data $D_{s}$ from the distribution approximated by *G*. So, using $D_{s}$, the synthetic data, instead of $D_{r}$, the real data, to train *C* cannot lead to gains in performance, because of the additional layer of approximation that *G* introduced.

Any improvements that one observes when using $D_{s}$ instead of $D_{r}$ are not due to systematic principles, but can only reflect artifacts (such as overfitting of *C* when using $D_{r}$) or random effects (implying that *C* may not be able to approximate the distribution when using $D_{r}$ as well as when using $D_{s}$). If the generator G is pre-trained on another dataset this upper limit does no longer exist.

In summary, it makes sense to evaluate the quality of generated data in terms of how much of the accuracy of the classifiers achieved on real data one can recover when replacing the real data with synthetic data. We perform this evaluation by establishing *recovery rate* $R(a_{r},a_{s})$ as per the following definition:


(2) $$R(a_{r},a_{s})=\frac{a_{s}}{a_{r}}$$

where $a_{r}$ is the test accuracy when a classifier is trained on the full real training data and $a_{s}$ is the test accuracy when the same classifier is trained on a synthetic dataset. Based on the above reasoning, one can expect that $R(a_{r},a_{s})\in [0,1]$, unless artifacts or random effects disturb the training processes in general.

Note already here that we demonstrate that the generated data do not merely reproduce the real data using other metrics (see just below for the definitions, and Section Experiments for the corresponding experiments).

#### 3.4.2 Nearest neighbour adversarial accuracy

It was shown that diffusion models, when provided with too little training data, tend to reproduce training data instead of generating fresh samples (Somepalli *et al.* 2023). To quantify at what rate we are affected by such effects, we follow the approach presented by Yale *et al.* (2020):


(3) $$\begin{array}{c} \begin{array}{c} AA_{\mathrm{truth}}=\frac{1}{n_{\mathrm{truth}}}\sum_{i=1}^{n_{\mathrm{truth}}}1(d_{\mathrm{truth},\mathrm{syn}}(i)>d_{\mathrm{truth},\mathrm{truth}}(i)) \end{array}\\ \begin{array}{c} AA_{\mathrm{syn}}=\frac{1}{n_{\mathrm{syn}}}\sum_{i=1}^{n_{\mathrm{syn}}}1(d_{\mathrm{syn},\mathrm{truth}}(i)>d_{\mathrm{syn},\mathrm{syn}}(i))\\ \mathrm{PrivacyLoss}=AA_{\mathrm{trut}h_{tr},\mathrm{syn}}-AA_{\mathrm{trut}h_{te},\mathrm{syn}} \end{array} \end{array}$$

where 1 is the indicator function. Scores of $AA_{\mathrm{truth}}=0.5$ and $AA_{\mathrm{syn}}=0.5$ mean that this metric cannot distinguish *syn* from *truth* (and vice versa). Scores closer to 0 reflect over-fitting, whereas scores closer to 1 reflect under-fitting. Using true training and held out test data $\mathrm{trut}h_{tr},\mathrm{trut}h_{te}$, one can compute privacy loss as $AA_{\mathrm{trut}h_{tr},\mathrm{syn}}-AA_{\mathrm{trut}h_{te},\mathrm{syn}}$. We do not report $AA_{\mathrm{truth},\mathrm{syn}}=\frac{1}{2}(AA_{\mathrm{truth}}+AA_{\mathrm{syn}})$ as in the original paper since underfitting on $AA_{\mathrm{truth}}$ and overfitting on $AA_{\mathrm{syn}}$ can mutually cancel each other, which leads to deceptively good scores despite the poor models that lead to these scores. For further details we refer the interested reader to the original paper (Yale *et al.* 2020).

#### 3.4.3 Classifier training

The most important question that we would like to answer is to what degree replacing true training data with synthetically generated training data leads to losses in prediction. For obtaining answers, we consider two scenarios.

First, we focus on predicting the prevalence of the genetic disease Amyotrophic Lateral Sclerosis (ALS), for which the first whole-genome based classifier was presented only recently (Luo *et al.* 2023). There, training data was selected from 10 405 individual genotypes, referring to 3192 cases, that is individuals affected by ALS, and 7213 controls, that is individuals not affected by ALS, as determined by medical professionals. Further, ALS is known to be a complex and hard to disentangle genetic disease, which means that reliable classification does not depend on a small set of genes. This was documented in Luo *et al.* (2023), by showing that good performance could only be established when using at least on the order of $10^{3}$ genes. Unlike in Luo *et al.* (2023), we train a common multilayer perceptron (MLP) as a binary (ALS or not) classifier. Note that performance rates achieved here exceed the performance rates displayed in Luo *et al.* (2023).

Second, we consider the 2504 genotypes provided through the 1000 Genomes (1KG) project (Auton *et al.* 2015) (stage 3), and the one of 26 population labels assigned to the genotypes, which gives rise to a classification task referring to one of 26 different labels. Again, we establish our primary classifier as an MLP, which parallels the situation for the ALS data.

To demonstrate that favorable usage of synthetically generated data does not depend on a particular type of classifier, we further experiment with a transformer based (“Transformer”) and a convolutional neural network (“CNN”) based classifier.

We evaluate each of the classifiers, trained with true data on the one hand, and synthetic data, as generated by the Diffusion Model, on the other hand, on held out test data (ALS: balanced, 520 positive/520 negative genotypes; 1KG: 10% of total data, unbalanced, haplotypes). We follow the experimental protocol presented in Luo *et al.* (2023) for the ALS data.

## 4 Experiments

In this chapter, we will evaluate the Diffusion Model according to the metrics outlined before. Furthermore, we denote the computational requirements for training the model and generating data samples in Table 2.

**Table 2.** Technical details for all generative models.^a^

	CNN	MLP	MLP + CNN	Transformer
Training time (s)	45.000	8.000	52.000	58.000
Parameter count	18.5561.68	310.094.848	328.651.401	135.280.130
Compute to generate one genome (Tflops)	1.73	0.62	2.35	49.29

a Tflops ($10^{12}$ flops) were determined using Pytorchs profiler. Training time was measured on a single Quadro 5000 RTX in seconds for the ALS dataset. Units are given in brackets ().

### 4.1 Training metrics

**Figure 3.**
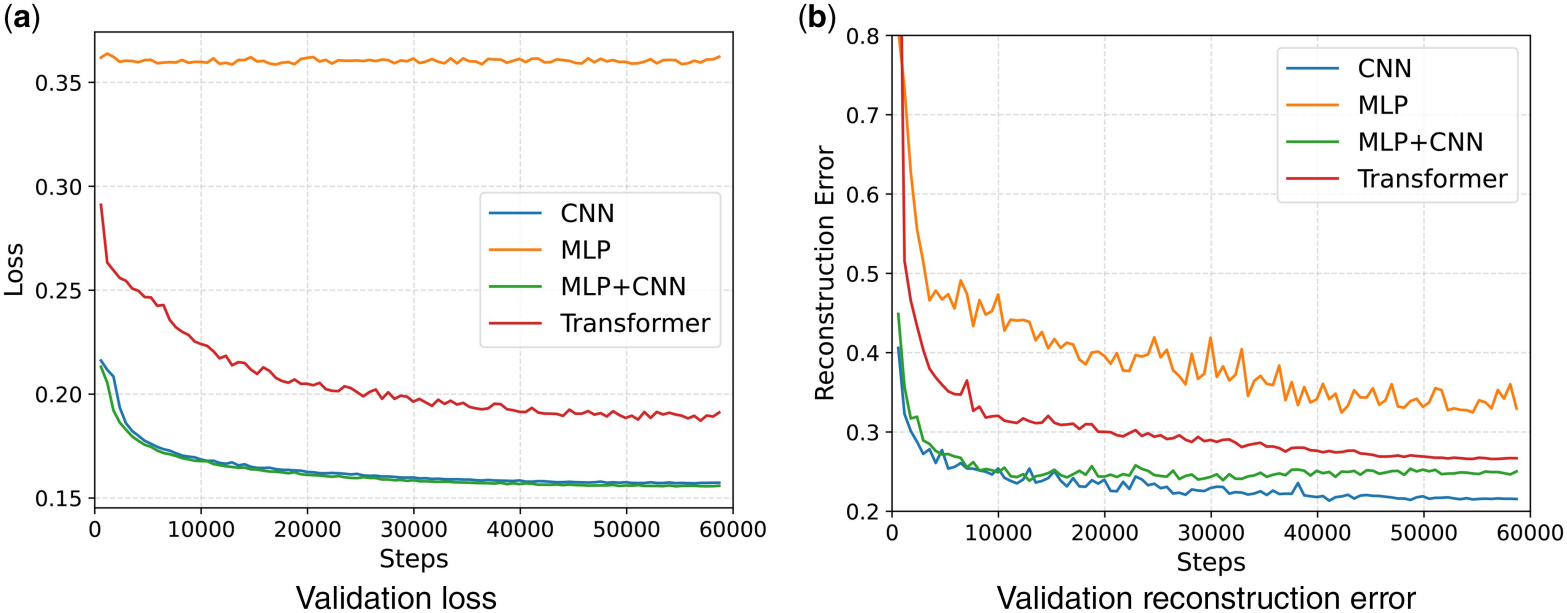
We display validation loss (a) and reconstruction error (b) e.g. $\left||x_{p}-x|\right|$ during training using single shot denoising for varying UNet architectures as part of the diffusion model. The validation loss in (a) decreases during training for all models but with different magnitudes, for the MLP model the decrease is negligible. In (b) the validation reconstruction error decreases evenly for all the models.

First, we show loss curves during training on validation data for the different diffusion model types, see Fig. 3. We observe that none of the models over-fit and all of them reduce the reconstruction error $\left||x-x_{p}|\right|$ ($x_{p}$ is the one shot denoising result, see the Appendix in the Supplementary Material for more details) as well as the loss continuously during training.

A closer look at the reconstruction error of the model, visualized in Fig. 3b, shows some interesting results. The MLP model performs only slightly worse compared to the CNN model in reconstruction error, and even though only small drops in loss during training can be observed for the MLP, the reconstruction error keeps improving. The Transformer based architecture seems to be performing well in terms of loss and reconstruction error. We note that reconstruction error is closely related to loss, but scaled by the noise schedule *t*, which means this error focuses more on large values of *t*. For further analysis we refer the interested reader to the Appendix in the Supplementary Material for a discussion of the diffusion process.

### 4.2 Evaluation

#### 4.2.1 Disease and population classification

First, we evaluate different classifiers trained on synthetic data in terms of recovering performance rates that one can achieve on true data—we recall that performance rates achieved on true data establish upper bounds for synthetic data generating mechanisms. See Table 3 for the corresponding results.

**Table 3.** Recovery rates on a hold out test set of true genotypes after training different ALS or 1KG population classifiers (MLP, Transformer or CNN) on different synthetically generated data types (generated by: MLP, Transformer, CNN, MLP + CNN).^a^

Classifier	CNN	MLP	MLP+CNN	Transformer
	ALS data			
MLP	71.51	**96.69**	94.26	73.77
Transformer	66.06	93.44	**93.89**	69.30
CNN	69.88	91.72	**93.17**	68.72
	1KG data			
MLP	15.58	65.80	**93.02**	13.28
Transformer	16.23	62.99	**84.98**	8.38
CNN	19.52	56.57	**77.54**	21.21
Average (all)	43.17	78.06	**89.48**	42.56

a The best synthetic data for each classifier type is marked in **bold**.

We observe that the combined MLP + CNN (U-Net) type architecture outperforms other generator architectures on both 1KG and ALS data, with near-perfect recovery rates on the ALS data. For ALS, the difference between MLP and MLP + CNN generated data is small, but on 1KG data, MLP + CNN clearly outperforms the other architectures. Synthetic data generated by Transformer and CNN based U-Net architectures perform poorly when used for training classifiers.

See the Appendix for the accuracy values on which recovery rates are based.

Secondly, we consider the (ubiquitous) scenario where a translational geneticist is forced to restrict her-/himself to a set of available true genotypes that is too small to train reliable classifiers. Here, we evaluate how augmenting small sets of real “seed” training data with larger amounts of synthetically generated data—which may be easily and cheaply available for the particular user—improves performance rates.

See Table 4 for the corresponding results. For example, augmenting only 5% of the real training data (leading to 70% accuracy when used in isolation for training) with synthetically generated data to the overall full amount of data nearly re-establishes the accuracy when using the full, real training dataset. This means that the availability of sufficiently large synthetic datasets may rescue efforts of researchers that remain with too little training datasets due to, e.g. budget constraints or restrictive access regulations.

**Table 4.** Accuracy improvements by integration of best synthetic data for best performing classification architecture.

	Amount real data	5%	10%	20%	50%
ALS Data	No syn data	70.96	76.50	80.90	84.60
	With syn data	84.83	85.01	85.34	85.70
1KG data	No syn data	29.01	43.99	71.52	85.19
	With syn data	83.98	86.93	87.11	87.50

#### 4.2.2 Nearest neighbour adversarial accuracy

We further evaluate the nearest neighbour adversarial accuracy and Privacy Loss (see Eq. 3), in Table 5. We observe that the combined MLP + CNN (U-Net) architecture performs well in terms of both nearest neighbour adversarial accuracy, and Privacy Loss. Of note, also Transformer and CNN generated data points deliver similar but slightly worse performance in terms of these metrics. We draw two conclusions from this:

**Table 5.** Result of nearest neighbour adversarial accuracy for generated datasets on the ALS and 1KG data; for AA the closer to 0.5 the better, for Privacy Loss the closer to 0 the better; best performance is **highlighted**.

			CNN	MLP	MLP + CNN	Transformer
ALS data	Test data	$AA_{\mathrm{truth}}$	0.735	0.255	**0.485**	0.92
		$AA_{\mathrm{syn}}$	0.68	1.0	0.93	**0.66**
	Train data	$AA_{\mathrm{truth}}$	0.81	0.005	**0.405**	0.93
		$AA_{\mathrm{syn}}$	**0.67**	1.0	0.92	0.69
		Privacy Loss	0.0325	0.125	0.0475	**0.02**
1KG data	Test data	$AA_{\mathrm{truth}}$	0.76	0.0	**0.63**	0.345
		$AA_{\mathrm{syn}}$	0.995	1.0	0.94	**0.92**
	Train data	$AA_{\mathrm{truth}}$	0.765	0.0	**0.385**	0.285
		$AA_{\mathrm{syn}}$	1.0	0.99	**0.74**	0.82
		Privacy Loss	0.05	**−0.005**	−0.2225	0.08

Interpreting the experiments, we conclude that the generated data is of sufficiently good quality, in particular that for the combined MLP + CNN architecture, documented by most scores being sufficiently close to 0.5. Improvements are conceivable, however, because the amount of training data used for training generators is likely at the lower limit of quantities required for sound estimation of such high dimensional and complex distributions.

To further quantify the preservation of privacy, we calculated L1, L2, and cosine distances between synthetic and real data points. Thereby, we can confirm that none of the synthetic data points matches any of the real data points. Note that this finding is crucial for maintaining the integrity of diffusion models in terms of privacy (i.e. diversity from a general perspective, which is also reflected in the NNAA score).

In summary, we conclude that the combined MLP + CNN U-Net architecture leads to synthetic genotypes/haplotypes that not only re-establish excellent performance in terms of classification, but also preserve the privacy of the real data used for training the generators to a sufficiently reliable degree.

## 5 Conclusion

In this work, we have presented, to the best of our knowledge, the first diffusion model based approach by which to generate full-length human genotypes. In this, by standard expansion of genotypes using human genome reference systems, we have also presented an approach by which to generate full-length human genomes at the level of DNA.

In our experiments, we have demonstrated, that the synthetically generated genotypes are realistic and that they do not just reproduce the real human genotypes used as input for training. When applying our approach to other datasets, we suggest re-consider the metrics by which to ensure the quality, privacy and diversity of the synthetic data.

To demonstrate the practical usefulness of synthetic genotypes, we have used synthetically generated genotypes as training data for disease- or population-related classifiers. We have shown that such practice re-establishes original performance rates to a degree that justifies their usage in translational genetics research.

Improvements of the diffusion models are readily conceivable by increasing the amount of training data. Note that although limited, one can expect amounts of training data available for training generative models to be larger in real world settings. The reason is that generators can be trained in safe environments, e.g. as part of the databases that host large numbers of genotype cohorts, which implies that none of the real data has to be shared. Including differential privacy mechanisms may further open up opportunities for usage of generative models in (e.g. federated learning) settings, where sharing parameters of the generative models may be beneficial.

## Author Contributions

Philip Kenneweg (Conceptualization, Methodology, Investigation, Formal analysis, Software, Writing – original draft, Writing – review & editing), Raghuram Dandinasivara (Conceptualization, Methodology, Writing – review & editing), Barbara Hammer (Conceptualization, Methodology, Supervision, Writing – review & editing), Alexander Schönhuth (Conceptualization, Methodology, Supervision, Writing – original draft, Writing – review & editing), Xiao Luo (Data curation, Software)

## Supplementary Material

btaf209_Supplementary_Data

## Data Availability

We publish all code at https://github.com/TheMody/GeneDiffusion.
