# Supplementary material for: Generating synthetic genotypes using diffusion models
Source: Bioinformatics. 2025 Jul 15;41(Suppl 1):i484–92. doi: 10.1093/bioinformatics/btaf209 (PMC12261458; doi:10.1093/bioinformatics/btaf209)
Supplement: btaf209_Supplementary_Data [file btaf209_supplementary_data.pdf]

Paper Generating Synthetic Genotypes using Diffusion Models:

Alt Text for Figures

Figure 1. Pre-processing pipeline

ALT TEXT: Diagram of a genomic data pre-processing pipeline. Shows genes with 5–100 SNPs each undergoing gene-specific PCA to reduce dimensionality. The final output is an 8-dimensional PCA vector per gene, forming an embedding of the full genome.

Figure 2. UnetMLP diffusion architecture

ALT TEXT: Diagram of the UnetMLP diffusion model architecture. Depicts input data passing through downsampling and upsampling blocks, with skip connections linking the two. Conditioning variables (class label y and timestep t) are injected into the upsampling blocks.

Figure 3a. Validation loss during training

ALT TEXT: Line plot showing validation loss over training steps for CNN, MLP, MLP+CNN, and Transformer-based diffusion models. All models show decreasing loss, with the MLP showing the least improvement.

Figure 3b. Reconstruction error during training

ALT TEXT: Line plot of validation reconstruction error over training steps. All models show declining error, with the CNN having the lowest error.

Alt Text for Tables

Table 1. Comparison with existing genome generators

ALT TEXT: Table comparing previous synthetic genome generation methods to the proposed diffusion model. Rows represent different models; columns list model type, data type, genome length, and conditioning. The proposed model is the only one generating full-length genotypes with conditioning.

Table 2. Technical details for generative models

ALT TEXT: Table showing training time, parameter count, and compute requirements for CNN, MLP, MLP+CNN, and Transformer diffusion models. MLP is fastest with fewest parameters; Transformer is most resource-intensive.

Table 3. Nearest Neighbour Adversarial Accuracy

ALT TEXT: Table reporting AAtruth, AAsyn, and Privacy Loss for each model on ALS and 1KG data. MLP+CNN achieves closest scores to optimal values with low privacy loss, indicating strong privacy and realism balance.

Table 4. Recovery rates for classifiers

ALT TEXT: Table of classifier accuracy recovery rates for ALS and 1KG tasks when trained on synthetic data. MLP+CNN-generated data consistently achieves highest recovery rates across classifier types.

Table 5. Accuracy with partial real + synthetic data

ALT TEXT: Table showing classifier accuracy on ALS and 1KG tasks using partial real data with and without synthetic augmentation. Shows that adding synthetic data significantly improves performance, approaching full-data accuracy.
